# Genetic Variations in Radiation and Chemotherapy Drug Action Pathways and Survival in locoregionally Advanced Nasopharyngeal Carcinoma Treated with Chemoradiotherapy

**DOI:** 10.1371/journal.pone.0082750

**Published:** 2013-12-10

**Authors:** Huai Liu, Bin Qi, Xiang Guo, Lin-Quan Tang, Qiu-Yan Chen, Lu Zhang, Ling Guo, Dong-Hua Luo, Pei-Yu Huang, Hao-Yuan Mo, Yan-Qun Xiang, Fang Qiu, Rui Sun, Ying Zhang, Ming-Yuan Chen, Yi-Jun Hua, Xing Lv, Lin Wang, Chong Zhao, Ka-Jia Cao, Chao-Nan Qian, Ming-Huang Hong, Hai-Qiang Mai

**Affiliations:** 1 State Key Laboratory of Oncology in South China, Guangzhou, P. R. China; 2 Department of Nasopharyngeal Carcinoma, Sun Yat-sen University Cancer Center, Guangzhou, P. R. China; 3 Department of Radiotherapy, Affilated Tumor Hospital of Guangzhou Medical College, Guangzhou, P. R. China; 4 Tumor Resources Bank, Sun Yat-sen University Cancer Center, Guangzhou, P. R. China; 5 Department of Epidemiology, Clinical Trial Study Center, Sun Yat-sen University Cancer Center, Guangzhou, P. R. China; MOE Key Laboratory of Environment and Health, School of Public Health, Tongji Medical College, Huazhong University of Science and Technology, China

## Abstract

**Background and Purpose:**

Treatment outcomes vary greatly in patients with nasopharyngeal carcinoma (NPC). The purpose of this study is to evaluate the influence of radiation and chemotherapy drug action pathway gene polymorphisms on the survival of patients with locoregionally advanced NPC treated with cisplatin- and fluorouracil-based chemoradiotherapy.

**Material and Methods:**

Four hundred twenty-one consecutive patients with locoregionally advanced NPC were prospectively recruited. We utilized a pathway approach and examined 18 polymorphisms in 13 major genes. Polymorphisms were detected using the LDR-PCR technique. Multifactor dimensionality reduction (MDR) analysis was performed to detect potential gene-gene interaction.

**Results:**

After adjustment for clinicopathological characteristics, overall survival was significantly decreased in patients with the *MPO* rs2243828 CT/CC genotype (HR=2.453, 95% CI, 1.687-3.566, *P*<0.001). The *ERCC1* rs3212986 CC (HR=1.711, 95% CI, 1.135-2.579, *P*=0.010), *MDM2* rs2279744 GT/GG (HR=1.743, 95% CI, 1.086-2.798, *P*=0.021), *MPO* rs2243828 CT/CC (HR=3.184, 95% CI, 2.261-4.483, *P*<0.001) and *ABCB1* rs2032582 AT/AA (HR=1.997, 95% CI, 1.086-3.670, *P*=0.026) genotypes were associated with poor progression-free survival. Prognostic score models based on independent prognostic factors successfully classified patients into low-, intermediate-, and high-risk groups. Furthermore, MDR analysis showed no significant interaction between polymorphisms.

**Conclusions:**

Four single nucleotide polymorphisms were associated with survival in patients with locoregionally advanced NPC treated with cisplatin- and fluorouracil-based chemoradiotherapy. Combining clinical prognostic factors with genetic information was valuable in identifying patients with different risk.

## Introduction

Prevalent in the south of China, nasopharyngeal carcinoma (NPC) has an ethnic and geographic distribution pattern that is distinctive from other head and neck cancers [1,2]. NPC is highly responsive to radiotherapy and systemic chemotherapy. Based on the results from several large prospective clinical trials using cisplatin- and fluorouracil (5-FU)-based chemoradiotherapy (CRT) [3-5], the combined treatment has become the standard regimen for locoregionally advanced NPC. However, even with this multimodal approach, approximately 30% of patients experience treatment failure within 5 years [4], and treatment outcomes vary widely, even among patients of the same clinical stage. Therefore, prospectively identifying patients who will have better or worse outcomes after CRT would aid in designing appropriate treatment strategies, highlighting the need for improved predictive markers.

The expression and activity levels of the critical enzymes related to radiation and chemotherapy drug response could affect treatment outcomes. Nucleotide excision repair (NER) and base excision repair (BER) are two important mechanisms involved in the repair of non-specific DNA damage induced by radiation and chemotherapy. The major enzymes involved are ERCC1, ERCC2 (NER), and XRCC1, hOGG1, APEX1, ADPRT (BER). Common genetic variations of above genes have been widely studied in the prognosis and susceptibility of various cancers [6-8]. For example, polymorphisms of the *XRCC1* gene, such as rs25487 (Arg399Gln), are capable of altering the phenotype of the XRCC1 protein, thus causing a deficiency in DNA repair [9] that is significantly related to patient survival [6]. 

Drug metabolism and transportation are important in the response of chemotherapy. MTHFR is a key enzyme in the folate metabolism pathway that regulates the intracellular folate pool for the synthesis and methylation of DNA [10]. High level of pre-treatment MTHFR expression was correlated with favorable response to fluorouracil-based chemotherapy [11]. GSTP1 is a primary enzyme responsible for the detoxification of platinum agents. It has been demonstrated to be a predictive marker of overall survival (OS) in cancer patients treated with cisplatin-based chemotherapy [12]. The *ABCB1* gene which was also known as *MDR1* gene encodes the multi-drug efflux pump P-glycoprotein (P-gp) which is involved in the transport of a wide range of anti-cancer drugs including cisplatin and fluorouracil [13]. The increase expression of ABCB1 was related with multidrug resistance and a poor response to chemotherapy for increased drugs efﬂux pathways [14]. Polymorphisms of these genes such as *MTHFR* rs1801131, *GSTP1* rs1138272 and *ABCB1* rs1045642 were associated with treatment outcomes in patients receiving chemotherapy using 5-Fu and cisplatin [6].

VEGF, as a critical angiogenic factor, plays an important role in cell growth and survival of endothelial cells and tumor cells. Moreover, VEGF production is a potential predictive marker for chemotherapy. It was significantly higher in cisplatin-resistant cancer cells than that in cisplatin-sensitive parental cells [15]. Genetic variation of *VEG*F was reported to be related with clinical outcome of patients treated with platinum-based chemotherapy [16]. The FGF-FGFR family also plays a critical role in cancer development because of its action in angiogenesis [17]. It has been demonstrated that *FGFR4* polymorphism is related with resistance to adjuvant therapy in primary breast cancer [18].

P53 plays an important role in cellular processes including cell-cycle arrest, DNA repair, and apoptotic cell death in response to cellular stress including chemotherapy [19]. MDM2 is a major negative regulator of p53. It directly binds to and inhibits p53 by regulating its location, stability, and activity as a transcriptional activator [20]. Previous study showed that *MDM2* polymorphism (rs2279744) located in the first intron of the *MDM2* promoter was an independent prognostic factor for cancer patients [21].

MPO is released by neutrophils and macrophages. It is a major enzyme involved in generating highly cytotoxic hypochlorous acid and other reactive oxygen species (ROS) [22], which result in oxidative stress-mediated apoptosis [23]. MPO is also extremely important in drug metabolism. With the ability to oxidize a wide variety of compounds and a broad range of functional groups [24], MPO may enhance the effect of chemotherapy. Functional polymorphism which influences the expression of MPO could impact on survival in patients receiving chemotherapy [25].

Because CRTs exert their effects through multistep, multigenic cascades, it is unlikely that any single SNP would have such a dramatic effect that it could serve as a sole predictive marker for response [6]. Therefore, in the current study, we use a pathway approach to investigate the impact of genetic variations in radiation and chemotherapy drug action pathway genes on the survival of patients with locoregionally advanced NPC treated with standard CRT. A comprehensive panel of 18 SNPs in 13 major genes involved in NER, BER, drug metabolism, drug disposition, oxidative stress reaction, the p53 pathway, *FGFR4* and *VEGF* were selected. We analyzed the association of each polymorphism with patient OS and progression-free survival (PFS) individually; then, according to multivariate analysis results, we built prognostic score models (PSMs) with genotypes and clinical characteristics to define different prognostic risk groups. Furthermore, the interaction between polymorphisms was also investigated.

## Materials and Methods

### Ethic statement

This study was approved by the independent Institute Research Ethics Committee at the Sun Yat-sen University Cancer Center (SYSUCC, Guangzhou, P. R. China), and written consents were obtained from all participants.

### Patient cohort

January 2002 and December 2005, 421 consecutive patients with locoregionally advanced NPC at the SYSUCC were enrolled in the study. All patients received standard CRT (details are described below). Patients were considered eligible if they had stage III or IVa-b [6th International Union against Cancer (UICC)/American Joint Committee on Cancer (AJCC) staging system] histological confirmed NPC. Other eligibility criteria included an age of 18 to 65 years, Han Chinese ethnicity, an Eastern Cooperative Oncology Group (ECOG) performance status of 0 or 1, WHO type II or III NPC, and treatment with concurrent CRT using cisplatin with or without sequential chemotherapy (induction or adjuvant). Patients who had other concomitant malignant diseases or who were previously treated with radiotherapy or chemotherapy were excluded.

### Treatment and follow-up

All patients were prospectively included in a disease-specific database. The pretreatment evaluation included a complete physical examination, magnetic resonance imaging (MRI) of the nasopharynx and neck region, fiber optic nasopharyngoscopy, chest X-ray, abdominal ultrasound, bone scan by emission computed tomography, complete blood count and liver and renal biochemistries. 

All patients received definitive radiotherapy with 6-MV photons. Uniform radiotherapy protocols for conventional two-dimensional radiotherapy (2D-CRT) and intensity-modulated radiotherapy (IMRT) at the SYSUCC were followed as previously described [26,27]. Radiotherapy was administered 5 times per week at 2 Gy/d for 2D-CRT and at 2.27 Gy/d for IMRT. The accumulated radiation doses were 68-72 Gy to the primary tumor, 60-62 Gy to the involved areas of the neck and 50 Gy to the uninvolved areas.

Concurrent chemotherapy was administered to all patients. Altogether, 28.7% and 25.9% of the patients received induction and adjuvant chemotherapy, respectively (Table 1). For induction chemotherapy, 2 cycles of PF chemotherapy were administered [cisplatin 100 mg/m^2^ i.v. drip on day 1 and 5-FU 1,000 mg/(m^2^·d) continuous i.v. for 120 hours, repeated every 3 weeks]. For concurrent chemotherapy, cisplatin was administered at 100 mg/m^2^ on days 1, 22, and 43 during radiotherapy or at 40 mg/m^2^ weekly during radiotherapy. For adjuvant chemotherapy, concurrent CRT followed by a combination of cisplatin (80 mg/m^2^) plus 5-FU [1000 mg/(m^2^·d)] was administered by 96-hour infusion every four weeks for three cycles.

**Table 1:** Demographic and clinical characteristics of patients.

**Patient characteristics**	***N*=421 (%)**
Age, years	
Median	45
Range	18-65
≤45y	215 (51.1)
>45y	206 (48.9)
Gender	
Male	322 (76.5)
Female	99 (23.5)
Histology	
WHO type II	20 (4.8)
WHO type III	401 (95.2)
T stage **^*a*^**	
T1	8 (1.9)
T2	139 (33.0)
T3	184 (43.7)
T4	90 (21.4)
N stage **^*a*^**	
N0	77 (18.3)
N1	156 (37.1)
N2	137 (32.5)
N3	51 (12.1)
Stage **^*a*^**	
III	284 (67.5)
IVa-b	137 (32.5)
EBV DNA	
<4000 copies/ml	235 (55.8)
≥4000 copies/ml	186 (44.2)
Treatment	
CCRT	191 (45.4)
IC+CCRT	121 (28.7)
CCRT+AC	109 (25.9)
RT technique	
2D-CRT	323 (76.7)
3D-CRT	14 (3.3)
IMRT	84 (20.0)
Follow-up time (months)	
Median	62.0
Range	5-125

^a^ 2002 American Joint Committee on Cancer/International Union Against Cancer staging system.

Abbreviation: CCRT, Concurrent Chemoradiotherapy; IC, Induction Chemotherapy; AC, Adjuvant Chemotherapy; RT, Radiotherapy; 2D-CRT, Conventional Two-Dimensional Radiotherapy; 3D-CRT, Three-Dimensional Conformal Radiotherapy; IMRT, Intensity-Modulated Radiotherapy.

Patients were assessed at the completion of treatment, at least once every three months over the first three years and at least once every six months thereafter. The patient evaluation included a clinical examination, nasopharyngeal endoscopy, MRI of the nasopharynx and neck area, chest X-ray and abdominal ultrasound. Follow-up ended on September 12, 2012, with a median follow-up time of 62.0 (range from 5 to 125) months (Table 1). During the follow-up period, 116 (27.6%) and 136 (32.3%) patients died or experienced disease progression, respectively. Five-year OS and PFS rates for the entire patient cohort were 74.3% and 67.8%, respectively.

### DNA extraction and genotyping

Blood for genotyping was prospectively collected at the time of enrollment. All sample collection and storage procedures were standardized. Genomic DNA was extracted from lymphocytes using the QIAamp DNA Blood Midi Kit (Qiagen, Hilden, Germany) according to the manufacturer’s protocol. The genes involved in drug action pathways were identified using the Pharmacogenomics Knowledge Base (http://www.pharmgkb.org/). The genes involved in radiation response pathways were identified by a search of the published literature. SNPs with a minor allele frequency of 0.05 or more in Asian populations were included for evaluation. Overall, we selected 18 SNPs from 13 major genes (Table 2). Genotyping of the SNPs was performed by the Shanghai BioWing Applied Biotechnology Company (BioWing, Shanghai, China) using the ligase detection reaction- polymerase chain reaction (LDR-PCR) technique [28,29]. To validate the genotyping results, 10% of the samples were randomly selected for genotyping by a second investigator; the agreement rate was 100%.

**Table 2:** Radiaton and chemotherapy pathway gene polymorphisms and survival in patients with NPC treated with cisplatin/5-FU based CRT.

**Pathway^*a*^**	**Gene**	**SNP**	**Genotype**	**Overall survival**				**Progression-free survival**			
				**Dead/Alive**	**HR (95%CI)^*b*^**	***P ^b^***	***Q***	**Failure/No**	**HR (95%CI) ^*b*^**	***P ^b^***	***Q***
R+D	*FGFR4*	rs351855									
			CC	32/81	Ref			40/73	Ref		
			CT	59/152	0.952 (0.704-1.288)	0.751		72/139	0.983 (0.668-1.448)	0.933	
			TT	21/68	0.824 (0.558-1.216)	0.330		22/67	0.680 (0.404-1.144)	0.680	
			CT/TT	80/220	0.916 (0.686-1.223)	0.551		94/206	0.890 (0.615-1.289)	0.539	
			Model-free test**^*c*^**			0.608	0.578			0.273	0.428
D+F	*ERCC1*	rs3212986									
			CC	78/196	Ref			104/170	Ref		
			CA	27/82	0.842 (0.542-1.306)	0.442		25/84	0.567 (0.366-0.878)	0.011	
			AA	8/24	1.049 (0.505-2.177)	0.898		7/25	0.597 (0.277-1.284)	0.186	
			CA/AA	35/106	0.882 (0.591-1.316)	0.538		32/109	0.573 (0.385-0.853)	0.006	
			Model-free test**^*c*^**			0.721	0.578			0.023	0.105
D+F	*ERCC2*	rs1799793									
			GG	104/263	Ref			123/244	Ref		
			AG	7/37	0.683 (0.317-1.470)	0.785		10/34	0.727 (0.381-1.387)	0.333	
			AA	1/4	1.315 (0.183-9.473)	0.329		1/4	0.823 (0.115-5.901)	0.846	
			AG/AA	8/41	0.726 (0.353-1.494)	0.384		11/38	0.735 (0.396-1.364)	0.329	
			Model-free test**^*c*^**			0.590	0.578			0.617	0.641
D+F	*ERCC2*	rs13181									
			TT	93/252	Ref			114/231	Ref		
			GT	17/47	1.067 (0.636-1.791)	0.806		18/46	0.871 (0.530-1.433)	0.587	
			GG	1/3	0.717 (0.100-5.151)	0.741		2/2	1.344 (0.331-5.454)	0.679	
			GT/GG	18/50	1.039 (0.627-1.721)	0.883		20/48	0.903 (0.562-1.453)	0.675	
			Model-free test**^*c*^**			0.915	0.596			0.783	0.715
R+D+F	*XRCC1*	rs25487									
			GG	57/173	Ref			68/162	Ref		
			AG	41/110	1.133 (0.759-1.693)	0.541		51/100	1.158 (0.805-1.665)	0.428	
			AA	13/20	1.797 (0.983-3.285)	0.057		14/19	1.587 (0.892-2.823)	0.116	
			AG/AA	54/130	1.243 (0.857-1.804)	0.251		65/119	1.230 (0.875-1.728)	0.234	
			Model-free test**^*c*^**			0.156	0.576			0.270	0.428
F	*MTHFR*	rs1801131									
			AA	55/166	Ref			65/156	Ref		
			AC	49/115	1.252 (0.851-1.840)	0.254		60/104	1.331 (0.937-1.892)	0.111	
			CC	8/21	1.148 (0.547-2.410)	0.716		9/20	1.117 (0.556-2.244)	0.756	
			AC/CC	57/136	1.236 (0.853-1.790)	0.263		69/124	1.298 (0.925-1.823)	0.131	
			Model-free test**^*c*^**			0.518	0.578			0.280	0.428
F	*MTHFR*	rs1801133									
			CC	47/145	Ref			55/137	Ref		
			CT	52/119	1.300 (0.876-1.930)	0.193		61/110	1.293 (0.898-1.863)	0.167	
			TT	12/39	0.948 (0.502-1.789)	0.857		18/33	1.286 (0.754-2.192)	0.356	
			CT/TT	64/158	1.216 (0.834-1.773)	0.310		79/143	1.291 (0.915-1.823)	0.146	
			Model-free test**^*c*^**			0.350	0.578			0.348	0.477
D+F	*ABCB1*	rs1045642									
			CC	44/106	Ref			48/102	Ref		
			CT	51/151	0.895 (0.597-1.343)	0.593		66/136	1.064 (0.734-1.543)	0.744	
			TT	19/48	1.083 (0.631-1.859)	0.771		22/45	1.064 (0.642-1.762)	0.811	
			CT/TT	70/199	0.940 (0.643-1.374)	0.748		88/181	1.064 (0.748-1.512)	0.730	
			Model-free test**^*c*^**			0.742	0.578			0.942	0.782
D+F	*ABCB1*	rs2032582									
			GG	30/78	Ref			35/73	Ref		
			GT	45/121	0.987 (0.621-1.567)	0.955		58/108	1.095 (0.720-1.666)	0.672	
			TT	20/70	0.756 (0.430-1.332)	0.334		22/68	0.682 (0.400-1.163)	0.160	
			AG	10/17	1.279 (0.623-2.623)	0.502		8/19	0.870 (0.403-1.878)	0.723	
			AT	7/11	1.608 (0.704-3.673)	0.259		10/8	2.398 (1.185-4.853)	0.015	
			AA	0/5				2/3	1.150 (0.276-4.785)	0.848	
			GT/TT/AG/AT/AA		0.953 (0.627-1.449)	0.822			0.996 (0.678-1.464)	0.984	
			Others vs. AT/AA		1.207 (0.560-2.598)	0.631			2.132 (1.176 -3.863)	0.013	
			Model-free test**^*c*^**			0.591	0.578			0.046	0.157
D	*MPO*	rs2243828									
			TT	63/245	Ref			71/237	Ref		
			CT	39/59	2.131 (1.428-3.178)	2.09E-4		53/45	2.779 (1.946-3.970)	1.91E-8	
			CC	11/1	8.994 (4.720-17.136)	2.42E-11		12/0	12.041 (6.457-22.454)	5.03E-15	
			CT/CC	50/60	2.561 (1.776-3.713)	7.14E-7		65/45	3.226 (2.302-4.521)	1.01E-11	
			Model-free test**^*c*^**			1.88E-11	2.08E-10			4.03E-17	5.52E-16
R	*hOGG1*	rs1052133									
			GG	39/106	Ref			44/101	Ref		
			CG	57/148	1.045 (0.695-1.572)	0.832		76/129	1.257 (0.886-1.825)	0.228	
			CC	16/49	0.899 (0.502-1.609)	0.720		15/50	0.725 (0.404-1.304)	0.283	
			CG/CC	73/197	1.009 (0.683-1.490)	0.964		91/179	1.121 (0.781-1.608)	0.536	
			Model-free test**^*c*^**			0.867	0.596			0.114	0.312
R	*APEX1*	rs1130409									
			TT	47/107	Ref			52/102	Ref		
			GT	51/151	0.824 (0.554-1.225)	0.338		64/138	0.962 (0.667-1.387)	0.835	
			GG	14/44	0.805 (0.443-1.462)	0.476		19/39	1.011 (0.597-1.709)	0.969	
			GT/GG	65/195	0.820 (0.563-1.193)	0.278		83/177	0.973 (0.688-1.376)	0.875	
			Model-free test**^*c*^**			0.582	0.578			0.970	0.782
R	*ADPRT*	rs1136410									
			TT	40/89	Ref			46/83	Ref		
			CT	51/156	0.796 (0.526-1.204)	0.280		63/144	0.834 (0.570-1.220)	0.349	
			CC	22/56	0.928 (0.552-1.562)	0.779		27/51	1.023 (0.636-1.646)	0.926	
			CC/CT	73/212	0.832 (0.565-1.223)	0.349		90/195	0.883 (0.618-1.260)	0.491	
			Model-free test**^*c*^**			0.544	0.578			0.541	0.618
D	*MDM2*	rs2279744									
			TT	18/80	Ref			21/77	Ref		
			GT	49/134	1.586 (0.923-2.722)	0.095		61/122	1.735 (1.056-2.851)	0.030	
			GG	46/89	2.041 (1.183-3.521)	0.010		54/81	2.127 (1.284-3.524)	0.003	
			GT/GG	95/223	1.778 (1.074-2.943)	0.025		115/203	1.899 (1.192-3.026)	0.007	
			Model-free test**^*c*^**			0.036	0.199			0.014	0.096
D	*VEGF*	rs2010963									
			GG	45/113	Ref			48/110	Ref		
			CG	47/146	0.877 (0.583-1.321)	0.530		61/132	1.069 (0.732-1.561)	0.729	
			CC	20/45	1.084 (0.640-1.836)	0.764		25/40	1.254 (0.773-2.035)	0.359	
			CG/CC	67/191	0.930 (0.637-1.358)	0.708		86/172	1.117 (0.784-1.591)	0.539	
			Model-free test**^*c*^**			0.687	0.578			0.655	0.641
D	*VEGF*	rs833061									
			TT	58/167	Ref			70/155	Ref		
			CT	49/124	1.144 (0.782-1.673)	0.489		60/113	1.159 (0.821-1.637)	0.401	
			CC	5/12	1.119 (0.448-2.794)	0.809		4/13	0.721 (0.263-1.975)	0.524	
			CT/CC	54/136	1.141 (0.788-1.673)	0.485		64/126	1.117 (0.796-1.568)	0.523	
			Model-free test**^*c*^**			0.783	0.578			0.519	0.618
D	*VEGF*	rs3025039									
			CC	79/212	Ref			100/191	Ref		
			CT	32/83	0.951 (0.630-1.435)	0.811		31/84	0.721 (0.481-1.079)	0.111	
			TT	1/7	0.387 (0.054-2.780)	0.345		3/5	0.957 (0.303-3.021)	0.941	
			CT/TT	33/90	0.883 (0.586-1.332)	0.553		34/89	0.482 (0.644-1.060)	0.095	
			Model-free test**^*c*^**			0.630	0.578			0.281	0.428

^a^ R, Radiation; D, DDP; F, 5-Fluorouracil

^b^ HR, 95%CI and P values were calculated by using Cox proportional hazard model with no adjustment.

^c^ The association analysis was done based on the genetic model-free test (2 degrees of freedom)

### Statistical analysis

The primary endpoint for this study was OS, defined as the time from the date of enrollment to the date of the last follow-up visit or death from any cause. The second endpoint was PFS, calculated from the date of enrollment to the date of the first failure at any site, death from any cause or last follow-up visit.

Clinicopathological characteristics were dichotomized as follows: age (≤45 y vs. >45 y), gender (male vs. female), histology (WHO type II vs. III), T stage (T1-2 vs. T3-4), N stage (N0-1 vs. N2-3), clinical stage (III vs. IVa-b), and plasma Epstein-Barr virus (EBV) DNA level (<4000 copies/ml vs. ≥4000 copies/ml). Associations of the genotypes with clinicopathological characteristics were evaluated by χ^2^ or Fisher’s exact tests. The Hardy-Weinberg equilibrium was determined for each SNP using a goodness-of-fit χ^2^ test. The impact of the polymorphisms on OS and PFS were examined using the Cox proportional hazard model with the calculation of hazard ratios (HRs) and 95% confidence intervals (CIs). To account for multiple comparisons in the SNP-based analysis, *Q* values set at 0.20 were computed using model-free test (two degrees of freedom) *P* values to quantify the probability that a P value may be a false positive [30], accepting a false discovery rate (FDR) of 20%. The survival end points were analyzed and estimated using the Kaplan-Meier method. The significance of the differences among survival curves was compared using the log-rank test. Multivariate analyses using the Cox proportional hazard model were used to detect independent prognostic factors, including genotypes and clinicopathological characteristics. The regression coefficient (“n” in the Cox regression equation HR=e^n^) of each independent prognostic factor was then transformed into an integral number to build a PSM [31]. We evaluated the predictive value of the PSMs and clinical stage by receiver operating characteristic (ROC) curve analysis [32]. The areas under curves (AUC) were compared between PSMs and overall stage.

To detect potential interactions among polymorphisms, the multifactor dimensionality reduction (MDR) analysis was performed (MDR software v3.0.2; available on http://sourceforge.net/projects/mdr/). The non-parametric MDR method is described in detail elsewhere [33,34]. Patients with missing data for polymorphisms were excluded from the analysis. We assumed that patients with beneficial or unfavorable genetic profiles have a survival much longer or shorter than the median. Therefore, we chose patients in the top and bottom quartiles of survival (OS and PFS, respectively) in the gene-gene interaction analysis to increase discriminating power [35,36]. In the interaction analysis, the ratio between patients in the top and bottom survival quartile for each genotype combination was evaluated. Combinations with more patients in top quartile than in the bottom quartile were considered high chance of favorable survival and vice versa. This procedure was carried out across 10-fold cross-validation samples to avoid over-fitting and was repeated for all possible combinations of two to four polymorphisms. The best combination was considered, if it had minimal prediction error and maximal cross-validation consistency (CVC). Statistical significance was further evaluated by a 1000-time permutation test to compare observed testing accuracies with those expected under the null hypothesis of no association (MDR permutation test module v1.0 beta 2; available on http://sourceforge.net/projects/mdr/files/mdrpt/).

All statistical tests were two-sided, and a P value of less than 0.05 was required for statistical significance. All the statistical analyses except those specifically mentioned were performed using SPSS 16.0 software (SPSS Inc., Chicago, IL).

## Results

### Patient characteristics and distribution of genotypes

Patient characteristics are presented in Table 1. For each SNP, two to seven samples could not be genotyped (98.1-99.5% call rate). Because only the C allele was detected for rs1138272 in our patient cohort, it was excluded from the analysis. Except for rs3212986 (*P*<0.001), rs1799793 (*P*=0.009), rs2032582 (*P*=0.020), rs2279744 (*P*=0.021) and rs833061 (*P*=0.021), all SNPs were tested at the Hardy-Weinberg equilibrium (*P*>0.05). 

### Polymorphisms and clinicopathological characteristics

Table S1 displays the associations between genotypes and clinicopathological characteristics. The distributions of *XRCC1* rs25478 and *MTHFR* rs1801133 were not in equilibration at overall stage (*P* =0.018) and EBV DNA level (*P* =0.043), respectively. For rs25478, genotype GG was significantly more frequently observed in patients with stage III (59.2%) vs. stage IV disease (47.7%). The rs1801133 genotype CC was more frequently observed in patients with high level of plasma EBV DNA than in patients with low EBV DNA (51.9% vs. 39.5%, respectively). 

### Polymorphisms and survival

Table 2 illustrates the associations between polymorphisms and survival in locoregionally advanced NPC patients. 

**Figure 1:**
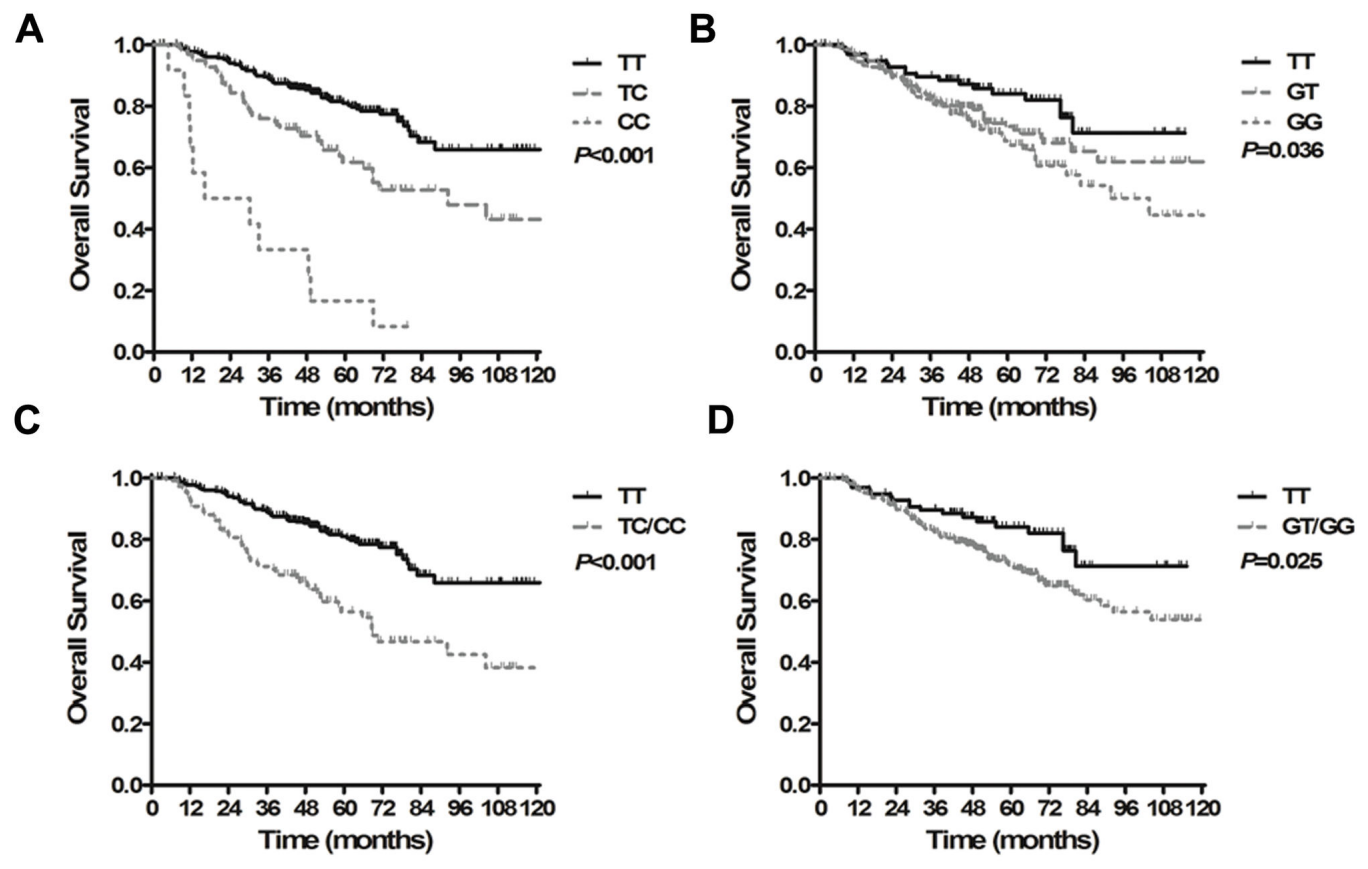
Kaplan-Meier curves for overall survival in patients with locoregionally advanced nasopharyngeal carcinoma treated with cisplatin- and fluorouracil-based chemoradiotherapy. (A) Survival time based on the MPO rs2243828 TT, CT and CC genotypes. (B) Survival time based on the MDM2 rs2279744 TT, GT and GG genotypes. (C) Survival time based on the MPO rs2243828 TT and CT/CC genotypes. (D) Survival time based on the MDM2 rs2279744 TT and GT/GG genotypes.

*MPO* rs2243828 and *MDM2* rs2279744 SNPs were significantly associated with patient OS (model-free test, *P*<0.001, Figure 1A and P=0.036, Figure 1B, respectively, Table 2). Patients with a variant C allele (CT/CC) at rs2243828 and a variant G allele at rs2279744 (GT/GG) had significantly increased death risks relative to those patients with the wild-type genotype (rs2243828: HR=2.561, 95% CI, 1.776-3.713, *P*<0.001, Figure 1C; rs2279744: HR=1.778, 95% CI, 1.074-2.943, *P*=0.025, Figure 1D). 

**Figure 2:**
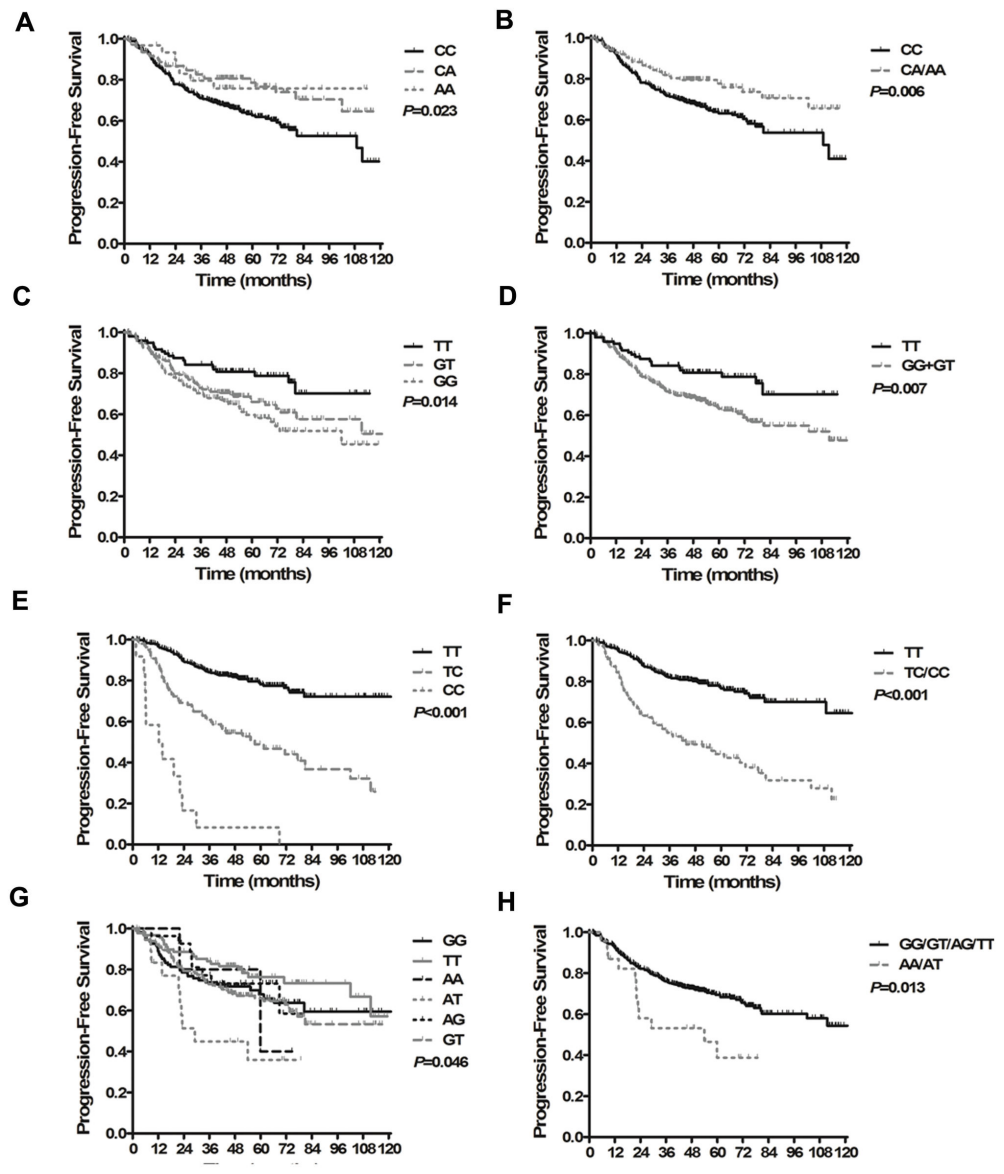
Kaplan-Meier curves for progression-free survival in patients with locoregionally advanced nasopharyngeal carcinoma treated with cisplatin- and fluorouracil-based chemoradiotherapy. (A) Survival time based on the ERCC1 rs3212986 CC, CA and AA genotypes. (B) Survival time based on the ERCC1 rs3212986 CC and CA/AA genotypes. (C) Survival time based on the MDM2 rs2279744 TT, GT and GG genotypes. (D) Survival time based on the MDM2 rs2279744 TT and GT/GG genotypes. (E) Survival time based on the MPO rs2243828 TT, CT and CC genotypes. (F) Survival time based on the MPO rs2243828 TT and CT/CC genotypes. (G) Survival time based on the ABCB1 rs2032582 GG, GT, TT, AG, AT and AA genotypes. (H) Survival time based on the ABCB1 rs2032582 GG/GT/TT/AG and AT/AA genotypes.

We found that the *ERCC1* rs3212986, *MDM2* rs2279744, *MPO* rs2243828 and *ABCB1* rs2032582 SNPs were significantly associated with patient PFS (model-free test, *P*=0.023 and Figure 2A, P=0.014 and Figure 2C, P<0.001 and Figure 2E, P=0.046 and Figure 2G, respectively). Carriers of the *ERCC1* rs3212986 (C8092A) genotypes CA/AA had significantly improved PFS compared with the wild-type homozygote CC (HR=0.573, 95% CI, 0.385-0.853, *P*=0.006, Figure 2B). Patients with the *MDM2* rs2279744 (SNP209TG) genotype GT/GG had a 1.8-fold greater progression risk than those patients with the TT genotype (HR=1.899, 95% CI, 1.192-3.026, *P*=0.007, Figure 2D). Compared to carriers of the *MPO* rs2243828 (T-764C) TT genotype, the 5-year PFS rate was significantly lower for CT/CC carriers (HR=3.226, 95% CI, 2.302-4.521, *P*<0.001, Figure 2F). Patients with the *ABCB1* rs2032582 AT/AA genotype had significantly increased progression risk relative to patients with other genotypes (GG/GT/TT/AG vs. AT/AA, HR=2.132, 95% CI, 1.176-3.863, *P*=0.013, Figure 2H). 

### Multivariate analysis

Multivariate analysis identified that age>45 y (HR=1.898, 95% CI, 1.293-2.785, *P*=0.001), N2-3 (HR=1.593, 95% CI, 1.068-2.376, *P*=0.022), EBV DNA≥4000 copies/ml (HR=1.579, 95% CI, 1.075-2.320, *P*=0.020) and *MPO* rs2243828 CT/CC genotype (HR=2.453, 95% CI, 1.687-3.566, *P*<0.001) were negative independent factors for OS (Table 3). This analysis also indicated that EBV DNA≥4000 copies/ml (HR=1.512, 95% CI, 1.059-2.160, *P*=0.023), *ERCC1* rs3212986 CC genotype (HR=1.711, 95% CI, 1.135-2.579, *P*=0.010), *MDM2* rs2279744 GT/GG genotype (HR=1.743, 95% CI, 1.086-2.798, *P*=0.021), *MPO* rs2243828 CT/CC genotype (HR=3.184, 95% CI, 2.261-4.483, *P*<0.001) and *ABCB1* rs2032582 AT/AA genotype (HR=1.997, 95% CI, 1.086-3.670, *P*=0.026) were negative independent factors for PFS (Table 3).

**Table 3:** Multivariate analysis of treatment outcomes.

**Variables**	**HR**	**95% CI**	***P***
*Overall survival*			
Age (≤45 vs. >45y)	1.898	1.293-2.785	0.001
Gender (Male vs. Female)	0.766	0.479-1.224	0.264
Histology (WHO type II vs. WHO type III)	1.441	0.581-3.574	0.431
T stage (T1-2 vs. T3-4)	1.238	0.832-1.840	0.292
N stage (N0-1 vs. N2-3)	1.593	1.068-2.376	0.022
EBV DNA level (<4000 vs. ≥4000 copies/ml)	1.579	1.075-2.320	0.020
*MPO* rs2243828 (TT vs. CT/CC)	2.453	1.687-3.566	<0.001
*MDM2* rs2279744 (TT vs. GT/GG)	1.550	0.930-2.582	0.092
*Progression-free survival*			
Age (≤45 vs. >45y)	1.040	0.738-1.464	0.823
Gender (Male vs. Female)	0.754	0.495-1.149	0.189
Histology (WHO type II vs. WHO type III)	0.944	0.456-1.955	0.878
T stage (T1-2 vs. T3-4)	1.204	0.840-1.725	0.313
N stage (N0-1 vs. N2-3)	1.415	0.980-2.045	0.064
EBV DNA level (<4000 vs. ≥4000 copies/ml)	1.512	1.059-2.160	0.023
*MPO* rs2243828 (TT vs. CT/CC)	3.184	2.261-4.483	<0.001
*ERCC1* rs3212986 (CA/AA vs. CC)	1.711	1.135-2.579	0.010
*MDM2* rs2279744 (TT vs. GT/GG)	1.743	1.086-2.798	0.021
*ABCB1* rs2032582 (others vs. AT/AA)	1.997	1.086-3.670	0.026

### Prognostic score models

**Figure 3:**
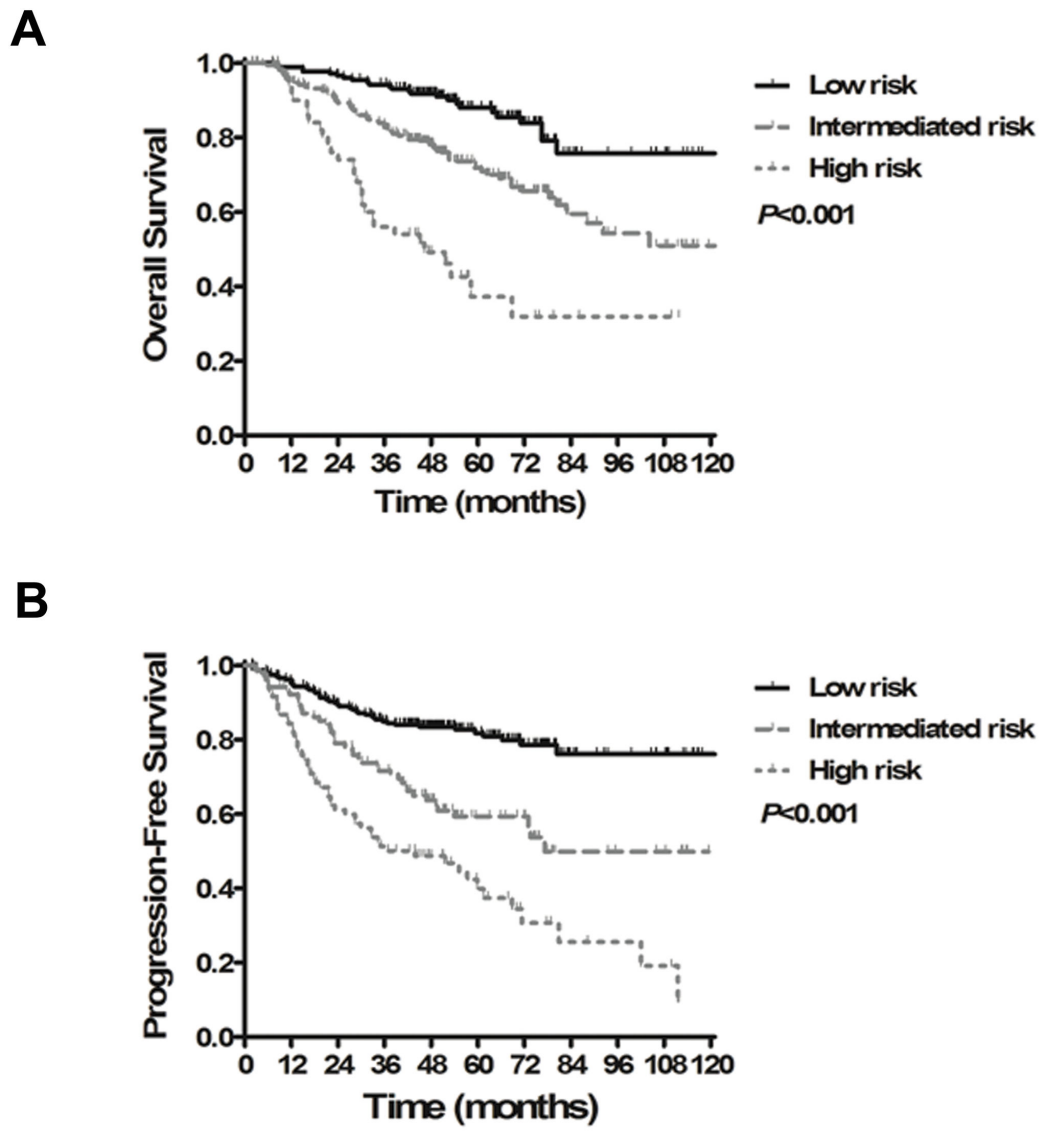
Kaplan-Meier survival curves for the three different risk groups classified by prognostic score models. (A) Overall survival. (B) Progression-free survival.

**Figure 4:**
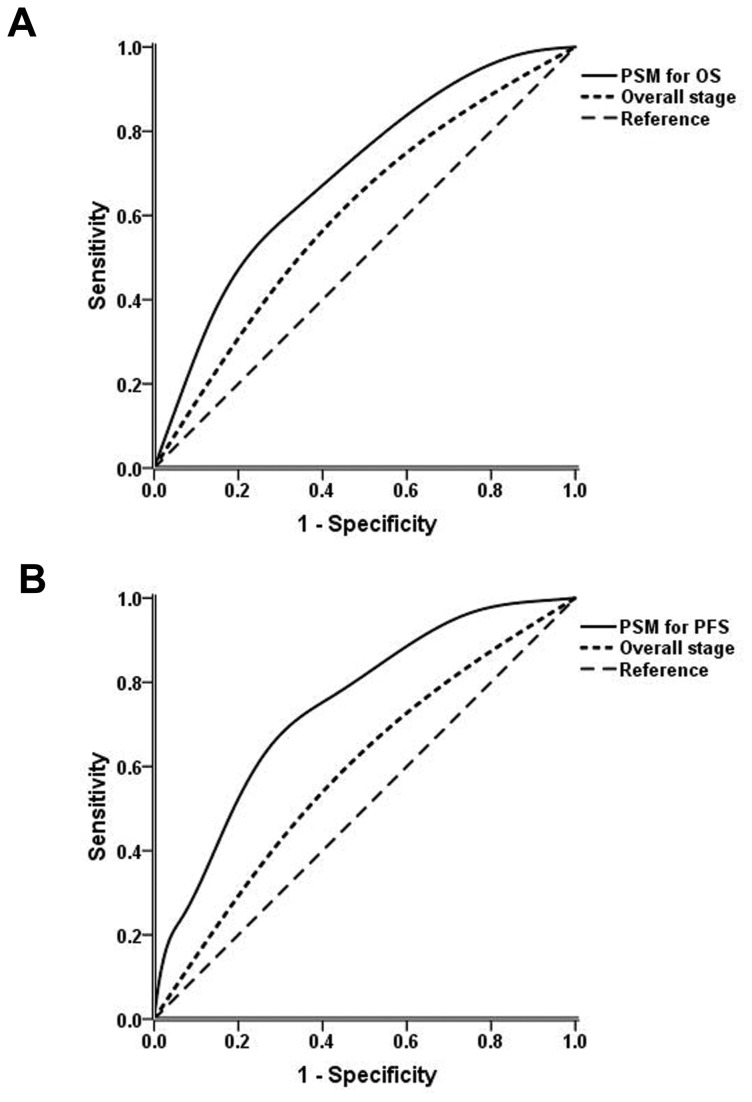
Receiver operating characteristic curves of prognostic score models (PSMs) and overall stage. (A) PSM and overall stage for overall survival. (B) PSM and overall stage for progression-free survival.

To build systemic PSMs for OS and PFS, an integral score was derived from the regression coefficients of each independent prognostic factor. If the factor was absent, a score of zero was recorded. If the factor was present, a score of 1 to 3 was recorded according to the n value (Table 4). The maximum scores for OS and PFS were 5 and 8, respectively. The overall score of the PSM for each patient was calculated as the total of the scores of each independent factor. All patients were then categorized into three groups according to cut-off points at the 25^th^ and 75^th^ percentiles of the score distribution as follows: low-risk group (total score 0-1 for OS, 0-2 for PFS), intermediate-risk group (total score 2-3 for OS, 3-4 for PFS) and high-risk group (total score 4-5 for OS, 5-8 for PFS). For OS, 235, 102 and 84 patients were in the low-, intermediate- and high-risk groups, respectively, with 5-year OS rates of 88.0%, 71.8% and 37.2%, respectively (*P*<0.001, Figure 3A). Referring to PFS, 176, 193 and 52 patients were in the low-, intermediate- and high-risk groups, respectively, and the 5-year PFS rates were 81.8%, 58.9% and 40.4%, respectively (*P*<0.001, Figure 3B). Figures 4A and 4B show the ROC curves for the PSMs and overall stage. The AUCs of the PSMs for OS and PFS were significantly larger than the AUCs for overall stage (OS: 0.702 vs. 0.572, respectively, *P*=0.002; PFS: 0.745 vs. 0.571, respectively, *P*<0.001) 

**Table 4:** Prognostic score models.

**Overall survival**			**Progression-free survival**		
**Factors**	**n (HR=e^n^)**	**Score**	**Factors**	**n (HR=e^n^)**	**Score**
Age (>45y)	0.64	1	EBV DNA (≥4000 copies/ml)	0.41	1
N stage (N2-3)	0.47	1	*MPO* rs2243828 (CT/CC)	1.16	3
EBV DNA (≥4000 copies/ml)	0.46	1	*ERCC1* rs3212986 (CC)	0.54	1
*MPO* rs2243828 (CT/CC)	0.90	2	*MDM2* rs2279744 (GT/GG)	0.56	1
			*ABCB1* rs2032582 (AT/AA)	0.69	2

### Multifactor dimensionality reduction analysis

We further performed the MDR analysis to evaluate potential interactions between polymorphisms. As shown in Table 5, the overall best MDR model was the one factor model using *MPO* rs2243828, which had a maximum CVC (10/10 for both OS and PFS) and minimum prediction error (43% for OS and 36% for PFS). Moreover, no potential interactions were found between investigated polymorphisms in this study (permutation test *P*>0.05 for the best two to four polymorphisms combinations).

**Table 5:** MDR analysis for the survival prediction in patients with locoregionally advanced nasopharyngeal carcinoma.

**The best combination**	**TBA**	**CVC**	***P* value ^*a*^**
***Overall survival***			
rs2243828	0.57	10/10	0.30
rs2243828, rs2032582	0.56	8/10	0.42
rs2032582, rs2279744, rs1136410	0.44	3/10	NR **^*b*^**
rs2032582, rs1136410, rs1801131, rs2010963	0.43	3/10	NR **^*b*^**
***Progression-free survival***			
rs2243828	0.64	10/10	0.06
rs2243828, rs1801131	0.62	6/10	0.11
rs2243828, rs1801131, rs2032582	0.53	6/10	0.67
rs2243828, rs2032582, rs2279744, rs351855	0.45	4/10	NR **^*b*^**

The above SNPs were specified as following: *MPO* rs2243828, *ABCB1* rs2032582, *MDM2* rs2279744, ADPRT rs1136410, *VEGF* rs2010963, MTHFR rs1801131, *FGFR4* rs351855.

*P* value for 1000-fold permutation test.^a^

*b* Negative result*, P* value nearly equals to 1.00.

Abbreviation: MDR, Multifactor dimensionality reduction; TBA, Testing balanced accuracy; CVC, Cross-validation consistency.

## Discussion

In this study, we used a pathway approach to systematically investigate the associations between the genotypes of 18 SNPs in 13 major genes from radiation and chemotherapy response pathways and survival in patients with locoregionally advanced NPC treated with standard CRT. Following adjustment for clinicopathological characteristics, we found that the *MPO* rs2243828 SNP was independently significant associated with OS and PFS and that the *ERCC1* rs3212986, *MDM2* rs2279744, and *ABCB1* rs2032582 SNPs were independently significant associated with PFS. Moreover, we performed MDR analysis and found no significant interaction between polymorphisms. We believe our results to be biologically plausible for the following evidences.

As mentioned above, MPO could oxidize a wide variety of compounds and a broad range of functional groups [24], then enhance the effect of chemotherapy. *MPO* rs2243828 (T-764C) is a functional promoter polymorphism that is linked with rs2333227 (G-463A) [6]. The wild-type alleles (T and G) are associated with higher expression levels of MPO [37,38]. Previous studies have found that low-activity *MPO* genotypes are associated with poor survival in esophageal and breast cancer patients treated with chemotherapy [6,25]. We also showed that patients with a variant allele had approximately 2.5-fold greater death risks and 3.2-fold greater progression risks than wild-type homozygous patients. To the best of our knowledge, this is the first study focusing on the relationship between the polymorphisms of *MPO* and NPC prognosis. However, some studies have found that the variant allele has a protective effect against cancer [39,40]. We hypothesize that this contradictory finding is a result of the variations in ROS activity during the different phases of cancer. In the early phases of carcinogenesis, ROS are important molecules that are involved in damaging normal cells and thus contribute to the induction of carcinogenesis. On the contrary, in the context of cancer treatment, ROS play a different role by contributing to tumor cell death mechanisms.

High expression of MDM2 has been detected in several cancers and is related to decreased response to treatment and poor prognosis [41]. *MDM2* rs2279744 (SNP309 T>G) is located in the first intron of the *MDM2* promoter and therefore has the potential to influence the expression of MDM2. Previous studies have shown that cells carrying the GG genotype have an increased binding affinity for the transcriptional activator Sp1, which subsequently results in higher expression levels of *MDM2* mRNA and protein and elicits a decreased response to DNA-damaging agents [42]. Several studies have also demonstrated that the GG genotype in patients correlates with poor survival [21]. Liu et al found that *MDM2* T309G (rs2279744) genotypes were not related with OS and PFS in patients with advanced non-small cell lung cancer (NSCLC) treated with platinum-based chemotherapy, however, the combined analysis showed that significant shorter survival was in patients with the *p53* Pro/Pro and *MDM2* GG genotype [43]. With respect to NPC, rs2279744 is associated with the risk of developing the disease [44]; however, no studies have focused on the clinical outcome of NPC related to this SNP. In the present study, we found that patients with the variant allele G had significant lower PFS than those patients with the wild-type homozygote. This association between genotypes and PFS could be explained by the attenuated activity of p53; this attenuation is related to the poor chemo/radio-sensitivity of the tumor [45,46].

The NER pathway is important in the repair of the DNA adducts which were typically caused by cytotoxic drugs such as cisplatin. ERCC1 is a major component of the NER complex and is the rate-limiting enzyme in the pathway. Previous studies have found that over expression of ERCC1 is associated with resistance to cisplatin-based chemotherapy in various cancers [47], including NPC [48]. A common polymorphism of the *ERCC1* gene, rs3212986 (C8092A) is located in the 3’ untranslated region and therefore may affect mRNA stability and result in a decreased expression level [49]. However, the association of the rs3212986 genotypes and cancer prognosis has been inconsistent. In the present study, we found that patients with the wild-type homozygous allele had significantly lower PFS rates than did those patients with CA/AA genotypes. Our result is consistent with study using cisplatin-based treatment for gastric cancers [50]. However, other studies have reported the opposite results in NSCLC and ovarian cancer patients who were treated with platinum-based chemotherapy [51,52]. These contradictory results may be ascribed to differences in patient populations, tumor characteristics or treatment combinations. Altough ERCC2 is another major component of NER, we did not find any association between *ERCC2* genotypes and survival in NPC patients. A previous study also found *ERCC2* polymorphism was not related with survival in NSCLC patients receiving platinum-based chemotherapy [53].

The *ABCB1* is closely related to clinical multidrug resistance. The mechanism has multiple aspects, including reduced drug accumulation, altered drug metabolism, increased tolerance of cellular damage and diminished apoptotic signaling [54]. The rs2032582 (G>T/A) SNP is located in exon 21 of the *ABCB1* gene and generates an amino acid change from Ala to Thr/Ser. This SNP is often at linkage disequilibrium with another SNP at exon 26, rs1045642 (3435 C/T) [55]. The phenotypes of both rs2032582 and rs1045642 are associated with the clinical outcomes of several cancers [56,57]. In the present study, we did not find any correlation between the rs1045642 genotypes and patient survival but did find that patients harboring the rs2032582 AT/AA genotypes had 2.13-fold greater progression risks than other genotypes. Although the exact biological reason is unclear, the change in activity of P-gp may account for the change in chemotherapy sensitivity. 

Additionally, we found that the *XRCC1* rs25478 and *MTHFR* rs1801133 genotypes were correlated with clinical stage and EBV DNA level, respectively. As mentioned above, XRCC1 is a key protein that is directly involved in the repair of DNA base damage, and the Arg399Gln amino acid variant may alter the phenotype of the XRCC1 protein, causing deficient DNA repair [9]. *XRCC1* Arg399Gln was reported to be associated with susceptibility and prognosis of various cancers [6,8].The rs1801133 (C667T) variant is a common polymorphism of the *MTHFR* gene that leads to an amino acid substitution and decreased enzyme activity [58]. A previous study showed that patients with the variant allele CT/TT have improved survival compared with patients with the CC allele [59]. However, the reasons for the association of these two SNPs with the identified NPC characteristics are still unclear and require intensive investigation.

Liu et al performed a multi-loci analysis in NSCLC patients and they found that interactions among *XRCC1* Arg194Trp, *XPC* PAT, *FAS* G-1377A, and *FASL* T-844C were associated with sensitivity to platinum-based chemotherapy [60]. However, the overall best MDR model was the one factor model using *MPO* rs2243828 and permutation test *P* values for the best two to four polymorphisms combinations were over 0.05 in the present study, revealing no significant interactions among polymorphisms.

PSMs were built for OS and PFS containing the significant SNPs described above. These PSMs categorized patients into three risk groups according to their prognosis and demonstrated certain value in predicting survival. 

There are some limitations to this study, however. First, as with any study of modest size, this study may lack a degree of generalizability. Second, 18 SNPs were examined in our study, which could lead to false results due to multiple comparisons; to address this concern, we used a *Q* value to maintain the FDR under 20%. Finally, our results require validation with a large patient cohort prior to clinical application. Therefore, an additional prospective multicenter study should be conducted to further validate our results within the NPC patient population.

In conclusion, this study used a pathway approach to demonstrate that genetic variations within *MPO*, *MDM2*, *ERCC1* and *ABCB1* were associated with survival in patients with locoregionally advanced NPC treated with cisplatin- and 5-FU-based CRT. Furthermore, our PSMs demonstrated that genetic polymorphisms in combination with clinical prognostic factors showed certain value in identifying patients from different risk groups. With prospective validation, our results have the potential to provide valuable information for individualized treatment.

## Supporting Information

Table S1 **Distribution of genotypes in patient clinical characteristics.**
(DOC)
