# Supplementary material for: Genetic Variations in Radiation and Chemotherapy Drug Action Pathways and Survival in locoregionally Advanced Nasopharyngeal Carcinoma Treated with Chemoradiotherapy
Source: PLoS One. 2013 Dec 10;8(12):e82750. doi: 10.1371/journal.pone.0082750 (PMC3858314; doi:10.1371/journal.pone.0082750)
Supplement: Table S1 — Distribution of genotypes in patient clinical characteristics. (DOC) [file pone.0082750.s001.doc]

**Table S1** **Distribution of genotypes in patient clinical characteristics**

| **Genotype** | **Age** | | **Gender** | | **T stage** | | **N stage** | | **Overall stage** | | **EBV DNA level**  **copies/ml** | |
| --- | --- | --- | --- | --- | --- | --- | --- | --- | --- | --- | --- | --- |
|  | **≤45y** | **>45y** | **Male** | **Female** | **T1-2** | **T3-4** | **N0-1** | **N2-3** | **III** | **IV** | **<4000** | **≥4000** |
| rs351855 |  |  |  |  |  |  |  |  |  |  |  |  |
| CC | 59 | 54 | 82 | 31 | 33 | 80 | 65 | 48 | 73 | 40 | 70 | 43 |
| CT | 104 | 107 | 164 | 47 | 83 | 128 | 114 | 97 | 153 | 58 | 114 | 97 |
| TT | 48 | 41 | 70 | 19 | 30 | 59 | 49 | 40 | 54 | 35 | 48 | 41 |
| *P* | 0.734 |  | 0.495 |  | 0.179 |  | 0.833 |  | 0.093 |  | 0.349 |  |
| rs3212986 |  |  |  |  |  |  |  |  |  |  |  |  |
| CC | 144 | 130 | 201 | 73 | 95 | 179 | 151 | 123 | 187 | 87 | 163 | 111 |
| CA | 55 | 54 | 86 | 23 | 43 | 66 | 61 | 48 | 71 | 38 | 56 | 53 |
| AA | 12 | 20 | 27 | 5 | 8 | 24 | 18 | 14 | 23 | 9 | 14 | 18 |
| *P* | 0.272 |  | 0.255 |  | 0.308 |  | 0.984 |  | 0.733 |  | 0.120 |  |
| rs1799793 |  |  |  |  |  |  |  |  |  |  |  |  |
| GG | 191 | 176 | 280 | 87 | 128 | 239 | 202 | 165 | 251 | 116 | 210 | 157 |
| AG | 21 | 23 | 35 | 9 | 16 | 28 | 26 | 18 | 29 | 15 | 20 | 24 |
| AA | 1 | 4 | 4 | 1 | 2 | 3 | 1 | 4 | 3 | 2 | 2 | 3 |
| *P* | 0.351 |  | 0.882 |  | 0.955 |  | 0.271 |  | 0.780 |  | 0.256 |  |
| rs13181 |  |  |  |  |  |  |  |  |  |  |  |  |
| TT | 179 | 166 | 265 | 80 | 125 | 220 | 185 | 160 | 233 | 112 | 193 | 152 |
| GT | 29 | 35 | 50 | 14 | 20 | 44 | 41 | 23 | 43 | 21 | 37 | 27 |
| GG | 4 | 0 | 2 | 2 | 1 | 3 | 2 | 2 | 2 | 2 | 2 | 2 |
| *P* | 0.099 |  | 0.381 |  | 0.730 |  | 0.287 |  | 0.787 |  | 0.960 |  |
| rs25487 |  |  |  |  |  |  |  |  |  |  |  |  |
| GG | 110 | 120 | 170 | 60 | 80 | 150 | 135 | 95 | 167 | 63 | 137 | 93 |
| AG | 83 | 68 | 121 | 30 | 50 | 101 | 79 | 72 | 90 | 61 | 80 | 71 |
| AA | 19 | 14 | 26 | 7 | 16 | 17 | 13 | 20 | 25 | 8 | 16 | 17 |
| *P* | 0.290 |  | 0.356 |  | 0.240 |  | 0.084 |  | 0.018 |  | 0.288 |  |
| rs1801131 |  |  |  |  |  |  |  |  |  |  |  |  |
| AA | 110 | 111 | 172 | 49 | 80 | 141 | 123 | 98 | 156 | 65 | 125 | 96 |
| AC | 87 | 77 | 124 | 40 | 58 | 106 | 89 | 75 | 110 | 54 | 93 | 71 |
| CC | 15 | 14 | 21 | 8 | 8 | 21 | 16 | 13 | 16 | 13 | 15 | 14 |
| *P* | 0.829 |  | 0.756 |  | 0.659 |  | 0.964 |  | 0.230 |  | 0.876 |  |
| rs1801133 |  |  |  |  |  |  |  |  |  |  |  |  |
| CC | 97 | 95 | 152 | 40 | 78 | 114 | 98 | 94 | 132 | 60 | 88 | 94 |
| CT | 86 | 85 | 129 | 42 | 51 | 120 | 98 | 73 | 115 | 56 | 104 | 67 |
| TT | 28 | 23 | 35 | 16 | 17 | 34 | 33 | 18 | 33 | 18 | 31 | 20 |
| *P* | 0.658 |  | 0.272 |  | 0.095 |  | 0.173 |  | 0.852 |  | 0.043 |  |
| rs1045642 |  |  |  |  |  |  |  |  |  |  |  |  |
| CC | 84 | 66 | 116 | 34 | 58 | 92 | 84 | 66 | 99 | 51 | 91 | 59 |
| CT | 97 | 105 | 150 | 52 | 65 | 137 | 117 | 85 | 140 | 62 | 106 | 96 |
| TT | 32 | 35 | 55 | 12 | 23 | 44 | 31 | 36 | 47 | 20 | 37 | 30 |
| *P* | 0.287 |  | 0.409 |  | 0.448 |  | 0.246 |  | 0.753 |  | 0.308 |  |
| rs2032582 |  |  |  |  |  |  |  |  |  |  |  |  |
| GG | 60 | 48 | 84 | 24 | 40 | 68 | 59 | 49 | 70 | 38 | 56 | 52 |
| GT | 78 | 88 | 123 | 43 | 65 | 101 | 91 | 75 | 113 | 53 | 87 | 79 |
| TT | 47 | 43 | 73 | 17 | 27 | 63 | 51 | 39 | 59 | 31 | 50 | 40 |
| AG | 14 | 13 | 18 | 9 | 7 | 20 | 20 | 7 | 19 | 8 | 21 | 6 |
| AT | 8 | 10 | 15 | 3 | 4 | 14 | 7 | 11 | 14 | 4 | 13 | 5 |
| AA | 4 | 1 | 4 | 1 | 3 | 2 | 3 | 2 | 3 | 2 | 4 | 1 |
| *P* | 0.568 |  | 0.600 |  | 0.308 |  | 0.305 |  | 0.902 |  | 0.075 |  |
| rs2243828 |  |  |  |  |  |  |  |  |  |  |  |  |
| TT | 158 | 150 | 236 | 72 | 104 | 204 | 179 | 129 | 207 | 101 | 175 | 133 |
| CT | 48 | 50 | 75 | 23 | 35 | 63 | 48 | 50 | 69 | 29 | 53 | 45 |
| CC | 6 | 6 | 10 | 2 | 7 | 5 | 4 | 8 | 8 | 4 | 6 | 6 |
| *P* | 0.992 |  | 0.944 |  | 0.225 |  | 0.086 |  | 0.823 |  | 0.817 |  |
| rs1052133 |  |  |  |  |  |  |  |  |  |  |  |  |
| GG | 63 | 82 | 106 | 39 | 52 | 93 | 89 | 56 | 98 | 47 | 85 | 60 |
| CG | 111 | 94 | 163 | 42 | 69 | 136 | 109 | 96 | 138 | 67 | 112 | 93 |
| CC | 37 | 28 | 49 | 16 | 26 | 39 | 32 | 33 | 46 | 19 | 37 | 28 |
| *P* | 0.081 |  | 0.365 |  | 0.642 |  | 0.173 |  | 0.868 |  | 0.757 |  |
| rs1130409 |  |  |  |  |  |  |  |  |  |  |  |  |
| TT | 76 | 78 | 122 | 32 | 54 | 100 | 91 | 63 | 106 | 48 | 86 | 68 |
| GT | 103 | 99 | 153 | 49 | 72 | 130 | 108 | 94 | 130 | 72 | 108 | 94 |
| GG | 32 | 26 | 43 | 15 | 19 | 39 | 31 | 27 | 42 | 16 | 38 | 20 |
| *P* | 0.751 |  | 0.649 |  | 0.921 |  | 0.538 |  | 0.440 |  | 0.264 |  |
| rs1136410 |  |  |  |  |  |  |  |  |  |  |  |  |
| TT | 69 | 60 | 107 | 22 | 43 | 86 | 70 | 59 | 83 | 46 | 71 | 58 |
| CT | 101 | 106 | 150 | 57 | 66 | 141 | 114 | 93 | 145 | 62 | 118 | 89 |
| CC | 40 | 38 | 61 | 17 | 36 | 42 | 45 | 33 | 52 | 26 | 44 | 34 |
| *P* | 0.700 |  | 0.082 |  | 0.070 |  | 0.886 |  | 0.542 |  | 0.939 |  |
| rs2279744 |  |  |  |  |  |  |  |  |  |  |  |  |
| TT | 51 | 47 | 79 | 19 | 34 | 64 | 61 | 37 | 68 | 30 | 61 | 37 |
| GT | 93 | 90 | 138 | 45 | 67 | 116 | 91 | 92 | 129 | 54 | 98 | 85 |
| GG | 66 | 69 | 101 | 34 | 45 | 90 | 77 | 58 | 84 | 51 | 73 | 62 |
| *P* | 0.887 |  | 0.534 |  | 0.829 |  | 0.113 |  | 0.270 |  | 0.335 |  |
| rs2010963 |  |  |  |  |  |  |  |  |  |  |  |  |
| GG | 82 | 76 | 119 | 39 | 58 | 100 | 90 | 68 | 112 | 46 | 91 | 67 |
| CG | 98 | 95 | 154 | 39 | 66 | 127 | 106 | 87 | 133 | 60 | 105 | 88 |
| CC | 32 | 33 | 44 | 21 | 22 | 43 | 34 | 31 | 37 | 28 | 38 | 27 |
| *P* | 0.934 |  | 0.133 |  | 0.864 |  | 0.809 |  | 0.115 |  | 0.774 |  |
| rs833061 |  |  |  |  |  |  |  |  |  |  |  |  |
| TT | 114 | 111 | 174 | 51 | 76 | 149 | 123 | 102 | 155 | 70 | 133 | 92 |
| CT | 89 | 84 | 130 | 43 | 64 | 109 | 100 | 73 | 113 | 60 | 92 | 81 |
| CC | 10 | 7 | 14 | 3 | 6 | 11 | 8 | 9 | 11 | 6 | 8 | 9 |
| *P* | 0.810 |  | 0.803 |  | 0.801 |  | 0.631 |  | 0.734 |  | 0.369 |  |
| rs3025039 |  |  |  |  |  |  |  |  |  |  |  |  |
| CC | 149 | 142 | 228 | 63 | 110 | 181 | 155 | 136 | 191 | 100 | 162 | 129 |
| CT | 58 | 57 | 85 | 30 | 33 | 82 | 71 | 44 | 83 | 32 | 67 | 48 |
| TT | 5 | 3 | 4 | 4 | 2 | 6 | 5 | 3 | 5 | 3 | 3 | 5 |
| *P* | 0.865 |  | 0.116 |  | 0.187 |  | 0.291 |  | 0.375 |  | 0.490 |  |
